# Laboratory Evaluation of Contact and Feeding Deterrent Effects of Selected Essential Oils Against Different Life Stages of *Cylas formicarius* (Coleoptera: Brentidae)

**DOI:** 10.3390/insects17060620

**Published:** 2026-06-12

**Authors:** Maria Jéssica dos Santos Cabral, Muhammad Haseeb, Otgonpurev Sukhbaatar, Marcus Alvarenga Soares

**Affiliations:** 1 Center for Biological Control, College of Agriculture and Food Sciences, Florida A&M University, Tallahassee, FL 32307, USA; jessica1.cabral@famu.edu; 2 Department of Chemistry, School of Applied Sciences, Mongolian University of Life Sciences, Ulaanbaatar 17024, Mongolia; otgonpurev@muls.edu.mn; 3 Departamento de Agronomia, Universidade Federal dos Vales do Jequitinhonha e Mucuri, Diamantina 39100000, MG, Brazil; marcus.alvarenga@ufvjm.edu.br

**Keywords:** alternative control, botanical pesticides, sweet potato weevil, pest management

## Abstract

The sweet potato weevil, *Cylas formicarius* (Fabricius) (Coleoptera: Brentidae), is one of the most destructive pests of sweet potato [*Ipomoea batatas* (L.) Lam.] crops worldwide. Current management of the sweet potato weevil relies heavily on conventional pesticides, raising concerns about pesticide residues, environmental impacts, and the development of pesticide resistance. This study evaluated the effects of seven essential oils (EOs): eucalyptus (*Eucalyptus globulus*), garlic (*Allium sativum*), marigold (*Calendula officinalis*), mustard seed (*Sinapis alba*), peppermint (*Mentha piperita*), rosemary (*Rosmarinus officinalis*), and thyme (*Thymus gobicus*) against different life stages of *C. formicarius* under laboratory conditions. Feeding activity, oviposition, larval mortality, pupal mortality, and adult survival were evaluated using EO concentrations of 1%, 5%, and 10%, with acetone and distilled water as control treatments. Each treatment consisted of six replicates, with 10 insects per replicate. Statistical analyses were performed using logistic regression models with a binomial distribution. Significant effects of oil type and concentration were observed as lethal to weevil larval and pupal stages. Similarly, the feeding and oviposition were significantly impacted (*p* < 0.0001). Peppermint oil exhibited the highest efficacy, causing complete or near-complete mortality of second- and third-instar larvae and pupae at 10%. This also substantially reduced adult survival, feeding activity, and oviposition. Rosemary, thyme, and eucalyptus, at higher concentrations. Most oils almost completely suppressed oviposition. These findings demonstrate that plant-derived essential oils (EOs) exhibit significant insecticidal activity against *Cylas formicarius*, indicating their promise as sustainable tools for integrated pest management programs in sweet potato production systems.

## Simple Summary

The sweet potato weevil is a serious pest that damages sweet potatoes and is difficult to control with conventional pesticides. Generally, this leads to pesticide residues and resistance. This study tested seven plant-based essential oils: eucalyptus, garlic, marigold, mustard seed, peppermint, rosemary, and thyme at three strengths (1%, 5%, and 10%) to examine their effects on weevil’s feeding, egg-laying, and survival. This study confirms that all tested essential oils reduced weevil feeding. Peppermint oil was the most effective treatment overall, causing high mortality of both larvae and pupae at all tested concentrations. Rosemary, thyme, and eucalyptus oils were moderately effective at the highest concentration evaluated (10%). Most essential oils, with the exception of garlic oil, significantly deterred female weevils from laying eggs. In adult weevils, mortality increased with higher concentrations of eucalyptus, garlic, marigold, peppermint, and thymus oils. Among the treatments tested, peppermint, rosemary, and thymus oils consistently demonstrated the greatest efficacy. These findings warrant further investigation under semi-field and field conditions to evaluate their potential for sustainable management of the sweet potato weevil.

## 1. Introduction

The sweet potato [*Ipomoea batatas* (L.) Lam.] is a versatile and nutritionally valuable crop widely cultivated for human consumption, animal feed, and industrial use [1]. Its tubers are rich in β-carotene, vitamin C, dietary fiber, and essential minerals, and serve as raw materials for alcohol, starch, and processed food production [1,2,3]. The crop exhibits remarkable adaptability, tolerating nutrient-poor soils and drought conditions, which makes it a reliable choice for farmers in diverse climates [1]. Over the past two decades, the role of sweet potatoes in improving nutrition and food security in developing countries has grown substantially [4], while their use in animal feed and industrial starch production has also increased [1]. Advances in sweet potato cultivation and breeding have improved yield and quality; however, production remains vulnerable to insect pests and diseases [5].

The sweet potato weevil, *Cylas formicarius* (Fabricius) (Coleoptera: Brentidae), is the most damaging pest of sweet potato crops worldwide, including in the United States, causing substantial economic losses both in terms of reducing yield and tuber quality [5,6]. Effective management of *C. formicarius* is therefore critical to sustaining sweet potato production [7]. Current pest management tools, including chemical insecticides, entomopathogenic fungi (*Beauveria bassiana*), pheromones, and entomopathogenic nematodes, are often considered insufficient to meet grower needs [8] to control this serious pest. This has intensified the search for alternative control options, particularly the applications of EOs (essential oils). Several EOs are promising candidates, offering insecticidal, antifeedant, oviposition deterrent, fumigant, larvicidal, pupicidal, and growth-disrupting properties, with minimal risk to non-target organisms and the environment [9,10,11,12,13].

Previous studies have demonstrated the potential of EOs as alternative tools for managing *C. formicarius* [14]. They reported strong insecticidal activity of the EO extracted from *Cleome serrata* against the sweet potato weevil, highlighting the bioactive potential of plant-derived compounds for sweet potato pest management [14]. EOs from several Lamiaceae species showed significant repellent activity against *C. formicarius*, with repellent efficacy increasing according to concentration [15]. These findings reinforce the growing interest in EOs as environmentally friendly alternatives to synthetic insecticides and support further investigation of their insecticidal, feeding deterrent, and oviposition-inhibiting effects against different developmental stages of *C. formicarius*.

This study aimed to evaluate stage-specific susceptibility, quantify concentration-dependent responses, and identify the most effective EOs for the larval and adult stages of *C. formicarius*.

## 2. Materials and Methods

### 2.1. Rearing and Maintenance of Insects

**Figure 1:**
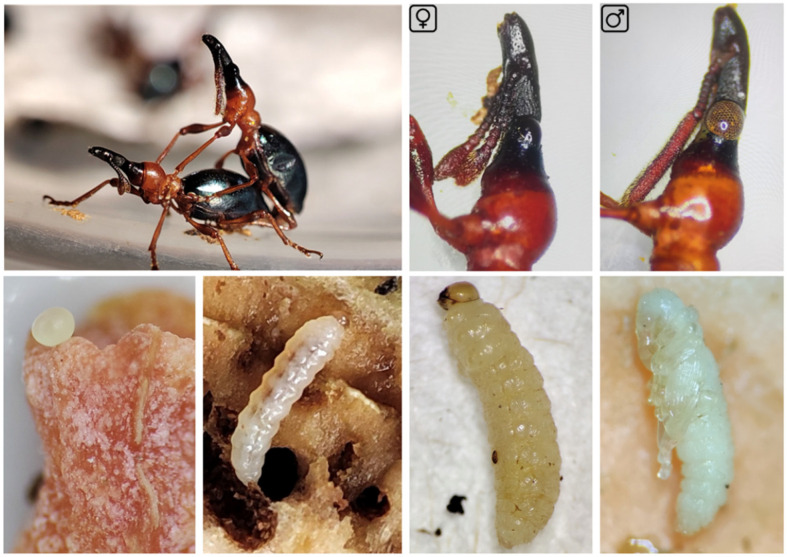
From left, first row: mating adults of *Cylas formicarius*, and female and male antennae club diagnostic characters. Second row from the left: an egg, second and third instar larvae, and a light-colored pupa.

The newly emerged male and female adults (1 day old), second- and third-instar larvae, and pupae of *C. formicarius* used in the experiments (Figure 1) were obtained from a laboratory colony maintained under controlled conditions (30 ± 5 °C temperature; 16 L:8 D photophase; 65 ± 5% RH) at the Center for Biological Control, College of Agriculture and Food Sciences, Florida A&M University, Tallahassee, FL, USA. The colony was continuously maintained on commercial sweet potato tubers (*Ipomoea batatas* var. Beauregard). These tubers served as food, oviposition, and rearing medium. The tubers were washed before use and replaced weekly, or earlier if they showed signs of desiccation, decay, or heavy infestation, thereby ensuring a constant supply of fresh substrate for the colony.

Adult weevils were collected daily after emergence, and male and female weevils were separated based on antennal club characters, then transferred to clean rearing containers supplied with fresh sweet potato tubers. The larvae of different instars and pupae were obtained by dissecting infested tubers under a stereomicroscope and separated according to morphological traits and body size. This protocol ensured the continuous availability of standardized insect life stages for use in bioassays.

### 2.2. Essential Oils and Solution Preparation

Seven EOs were evaluated: eucalyptus (*Eucalyptus globulus*), garlic (*Allium sativum*), marigold (*Calendula officinalis*), white mustard (*Sinapis alba*), peppermint (*Mentha piperita*), rosemary (*Rosmarinus officinalis*), and thyme (*Thymus gobicus*), and active compounds of the EOs (Table 1). These EOs (analytical grade; 100% pure) were purchased from Amazon and stored in their original bottles under refrigeration (±4 °C) to prevent degradation by light or temperature before use. Analytical-grade acetone was used for dilution of the EOs. This was obtained from Fisher Chemical.

The EOs used in this study were commercially obtained natural plant extracts containing the characteristic mixture of volatile bioactive compounds of each species. No synthetic formulations or isolated analytical-grade compounds were used. The oils were utilized according to the manufacturers’ specifications.

After homogenization (manual shaking) in 50 mL Falcon tubes, the solutions were stored in amber glass bottles with screw caps to prevent acetone evaporation and volatilization of active compounds. All solutions were prepared immediately before the bioassays to ensure chemical stability. Two additional treatments were used as controls: Control 1, distilled water, to evaluate the effect of an aqueous solution without solvents or active compounds; and Control 2, pure acetone (100%), to verify possible solvent effects on the tested insects (Table 2).

### 2.3. Feeding Activity of Cylas formicarius

Commercial sweet potato tubers were purchased from Walmart in Tallahassee, FL, USA. Before preparing the sweet potato disks, the roots were thoroughly washed with water to remove possible surface residues and contaminants. Then, they were cut into uniform disks of approximately 3 cm in diameter × 5 cm in thickness using a sterilized scalpel to avoid contamination. Each disk was immersed in one of the EO solutions (1%, 5%, or 10%) or in the control treatments (distilled water or 100% acetone) for 10 s. After immersion, the disks were dried at room temperature on sterilized filter paper until complete evaporation of the solvent.

One-day-old starved adults of *C. formicarius* were used in the bioassays. For each replicate, 10 vigorous insects (5 males and 5 females) were placed in Petri dishes (9.5 × 1.5 cm) containing one treated sweet potato disk. Insects were handled with fine-bristle brushes to avoid physical damage. To prevent disk desiccation and fungal growth during the experimental period, each disk was placed on filter paper moistened with water. The Petri dishes were maintained under controlled conditions (30 ± 5 °C temperature; 16 L:8 D photophase; 65 ± 5% RH).

After 24 h of exposure, food consumption was evaluated by directly counting the number of circular perforations on the disk surface using a stereomicroscope. Each treatment was replicated six times, totaling 60 insects per treatment. Data were subjected to analysis of variance (ANOVA) in a completely randomized design, considering insect sex, EO type, and concentration as factors. When significant differences were detected (*p* ≤ 0.05), means were compared using Tukey’s test (α = 0.05).

### 2.4. Evaluation of Larval Survival

The sweet potato tubers were cut into cubes of approximately 0.5 × 0.5 cm, which were immersed in EO solutions and control treatments, and subsequently air-dried at room temperature. Then, 10 s and third-instar larvae of *C. formicarius* were carefully introduced into cavities within the sweet potatoes. Larval mortality was assessed every 24 h via direct inspection under a stereomicroscope. Larvae were considered dead when no movement was observed following gentle mechanical stimulation with a fine-bristled brush. Each replicate consisted of 10 larvae, for a total of six replicates per treatment.

### 2.5. Evaluation of Pupal Survival

For the pupal assays, the sweet potato tubers were cut into cubes of 0.5 × 0.5 cm, immersed in EO solutions or control treatments, and then air-dried at room temperature. After preparation, 10 pupae of *C. formicarius* were carefully inserted individually into pre-drilled cavities in the treated cubes to avoid mechanical damage. Pupal mortality was monitored every 24 h for up to 7 days, with individuals considered dead if they failed to emerge as adults by the end of the experiment. Each replicate consisted of 10 pupae, with a total of six replicates per treatment.

### 2.6. Oviposition Assay

The sweet potato slices measuring approximately 3 cm in diameter × 0.5 cm in thickness were prepared, immersed in EO solutions or control treatments for 30 s, air-dried at room temperature on sterilized filter paper, and then placed individually in Petri dishes (9.5 × 1.5 cm). Then, 10 females and 10 males of *C. formicarius* (1 day old), obtained from the laboratory colony, were introduced into each dish to allow mating and oviposition under the controlled conditions. After 24 h of exposure, the treated slices were carefully removed and examined under a stereomicroscope, and the total number of eggs deposited on each slice was recorded. Each treatment was replicated six times, resulting in a total of 60 females evaluated per treatment.

### 2.7. Adult Survival Assay

Adult survival was evaluated following the feeding and exposure protocol described in Section 2.3. For each replicate, 10 vigorous *C. formicarius* adults (5 males and 5 females, 1 day old) were transferred to Petri dishes (9.5 × 1.5 cm) containing the sweet potato disks previously treated with EO solutions and controls. The disks were placed on filter paper moistened with water to prevent desiccation and were replaced every 48 h to prevent deterioration. Adult mortality was monitored every 24 h via direct observation under a stereomicroscope. Individuals of the weevil were considered dead if they exhibited no movement following gentle stimulation with a fine-bristled brush. Monitoring continued for up to 8 days. Each treatment was replicated six times, totaling 60 adults (30 males and 30 females) per treatment.

### 2.8. Statistical Analysis

The mortality and survival data were analyzed using logistic regression models with binomial distribution and logit link function, as recommended for discrete response variables and experiments with relatively small sample sizes. The number of dead or surviving insects relative to the total number of insects per experimental unit was used in the analyses. Abbott’s correction was not applied because control mortality remained below the threshold generally considered to require correction and did not substantially affect the interpretation of treatment effects. EO type, concentration, and their interaction were included as fixed effects in the models. The significance of treatment effects was evaluated using the likelihood ratio under the chi-square test. When significant differences were detected, comparisons among treatments were performed using estimated marginal means at *p* < 0.05. Each Petri dish containing 10 insects was considered one experimental unit, with five replicates per treatment. Individual insects were not treated as independent replicates to avoid pseudo-replication. Oviposition and feeding data were summarized as mean ± standard error (SE). All statistical analyses were performed using statistical analyses were performed using SAS version 9.4 (SAS Institute Inc., Cary, NC, USA).

## 3. Results

### 3.1. Feeding Activity of Cylas formicarius

**Figure 2:**
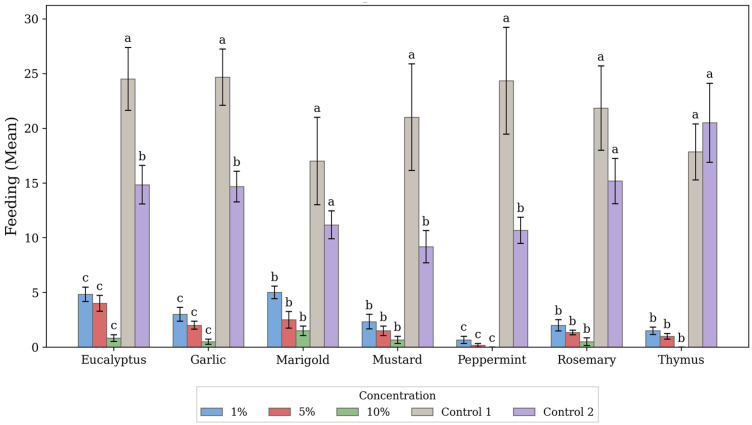
Feeding activity of *Cylas formicarius* on sweet potato disks treated with seven essential oils (eucalyptus, garlic, marigold, mustard, peppermint, rosemary, and thyme) at three concentrations (1%, 5%, and 10%). Control 1 = water; Control 2 = acetone. Bars represent mean ± standard error (SE) (n = 5). Different letters above the bars indicate significant differences among treatments, as determined by logistic regression followed by estimated marginal mean comparisons at *p* < 0.05.

Feeding activity differed significantly among EO treatments and concentrations (Figure 2). Logistic regression analysis revealed significant effects of oil type (LR χ^2^ = 37.98; df = 6; *p* < 0.0001) and concentration (LR χ^2^ = 804.38; df = 4; *p* < 0.0001) on feeding response, whereas the interaction between oil type and concentration was not significant (LR χ^2^ = 19.33; df = 24; *p* = 0.7343).

The control treatments presented the highest feeding means across all evaluated EOs. Control 1 showed the greatest feeding activity, with mean values ranging from 17.0 ± 3.9 to 24.5 ± 2.9, whereas Control 2 ranged from 9.2 ± 1.5 to 20.5 ± 3.6. In contrast, feeding was substantially reduced in the EO treatments, particularly at the 10% concentration, which consistently had the lowest feeding means.

For eucalyptus, feeding decreased from 4.8 ± 0.7 at 1% to 0.8 ± 0.4 at 10%, while the controls presented significantly higher values (24.5 ± 2.9 and 14.8 ± 1.7, respectively). Similar trends were observed for garlic, mustard, peppermint, rosemary, and thymus oils, all of which demonstrated strong reductions in feeding at higher concentrations. Peppermint oil at 10% exhibited one of the lowest feeding means observed in the study (0.1 ± 0.1). The results demonstrated that increasing the EO concentration significantly reduced the feeding activity of *C. formicarius*, with the 10% concentration generally producing the strongest inhibitory effect across all evaluated oils.

### 3.2. Second-Instar Larval Mortality

**Figure 3:**
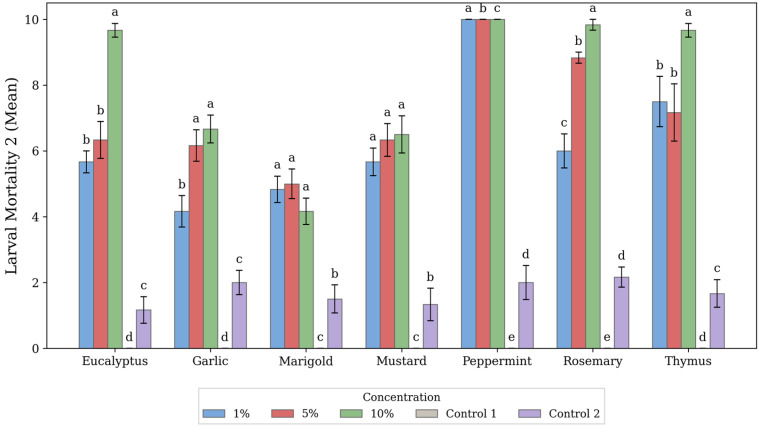
Mortality of second-instar larvae of *Cylas formicarius* exposed to seven essential oils (eucalyptus, garlic, marigold, mustard, peppermint, rosemary, and thyme) at three concentrations (1%, 5%, and 10%). Control 1 = water; Control 2 = acetone. Bars represent mean larval mortality ± standard error (SE) (n = 5). Different letters above the bars indicate significant differences among treatments, as determined by logistic regression followed by estimated marginal mean comparisons at *p* < 0.05.

Second-instar larval mortality differed significantly among EO treatments and concentrations (Figure 3). Logistic regression analysis revealed significant effects of oil type (LR χ^2^ = 162.60; df = 6; *p* < 0.0001), concentration (LR χ^2^ = 1117.72; df = 4; *p* < 0.0001), and the interaction between oil type and concentration (LR χ^2^ = 119.14; df = 24; *p* < 0.0001).

Larval mortality increased with increasing EO concentration, with the 10% concentration generally producing the highest mortality levels. Peppermint oil exhibited the strongest insecticidal activity, reaching 100% mortality at all evaluated concentrations (10.0 ± 0.0). Rosemary and thymus oils also caused high mortality, particularly at 10%, with mean mortalities of 9.8 ± 0.2 and 9.6 ± 0.3, respectively.

Eucalyptus and garlic oils demonstrated moderate to high larval mortality, with mortality increasing from 5.7 ± 0.4 and 4.2 ± 0.5 at 1% to 9.7 ± 0.2 and 6.7 ± 0.4 at 10%, respectively. Marigold and mustard oils presented comparatively lower mortality levels, particularly at lower concentrations. In contrast, control treatments exhibited minimal larval mortality, with Control 1 showing values near zero and Control 2 ranging from 1.1 ± 0.4 to 2.2 ± 0.3. These results showed that the higher concentrations of EOs significantly increased mortality of second-instar larvae of *C. formicarius*, with peppermint, rosemary, and thymus oils showing the greatest insecticidal effects.

### 3.3. Third-Instar Larval Mortality

**Figure 4:**
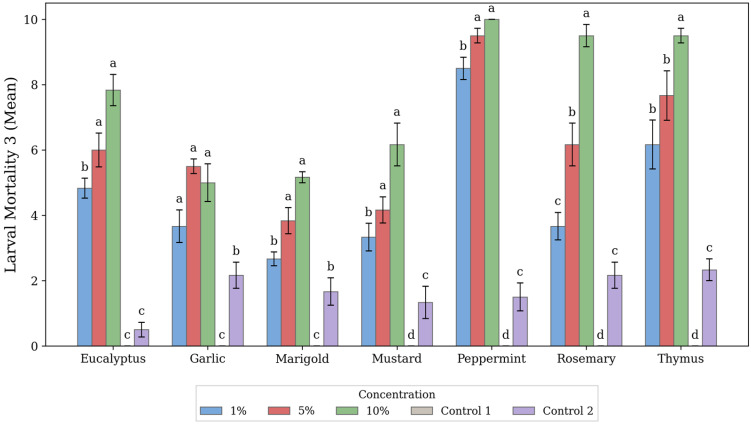
Mortality of third-instar larvae of *Cylas formicarius* exposed to seven essential oils (eucalyptus, garlic, marigold, mustard, peppermint, rosemary, and thyme) at three concentrations (1%, 5%, and 10%). Control 1 = water; Control 2 = acetone. Bars represent mean larval mortality ± standard error (SE) (n = 6). Different letters above the bars indicate significant differences among treatments, as determined by logistic regression followed by estimated marginal mean comparisons at *p* < 0.05.

Third-instar larval mortality was found to be significantly different among EO treatments and concentrations (Figure 4). Logistic regression analysis revealed significant effects of oil type (LR χ^2^ = 161.95; df = 6; *p* < 0.0001), concentration (LR χ^2^ = 903.17; df = 4; *p* < 0.0001), and the interaction between oil type and concentration (LR χ^2^ = 87.63; df = 24; *p* < 0.0001). As observed for second-instar larvae, mortality generally increased with increasing EO concentration. Peppermint oil again showed the highest efficacy, reaching 100% mortality at 10% (10.0 ± 0.0) and maintaining high mortality even at lower concentrations. Rosemary and thymus oils also caused elevated larval mortality, particularly at 10%, with values of 9.5 ± 0.4 and 9.5 ± 0.2, respectively.

Eucalyptus oil increased larval mortality from 4.8 ± 0.4 at 1% to 7.8 ± 0.5 at 10%, while garlic oil showed intermediate mortality levels ranging from 3.7 ± 0.5 to 5.0 ± 0.6 across concentrations. Marigold and mustard oils produced moderate mortality responses, especially at 10%, with means of 5.2 ± 0.2 and 6.2 ± 0.7, respectively. Control treatments showed low mortality values throughout the experiment, with Control 1 remaining close to zero and Control 2 ranging from 0.5 ± 0.2 to 2.3 ± 0.4. Overall, the results indicated that EO concentration significantly affected third-instar larval mortality, with peppermint, rosemary, and thymus oils providing the strongest insecticidal activity against *C. formicarius* larvae.

### 3.4. Pupal Mortality of Cylas formicarius

**Figure 5:**
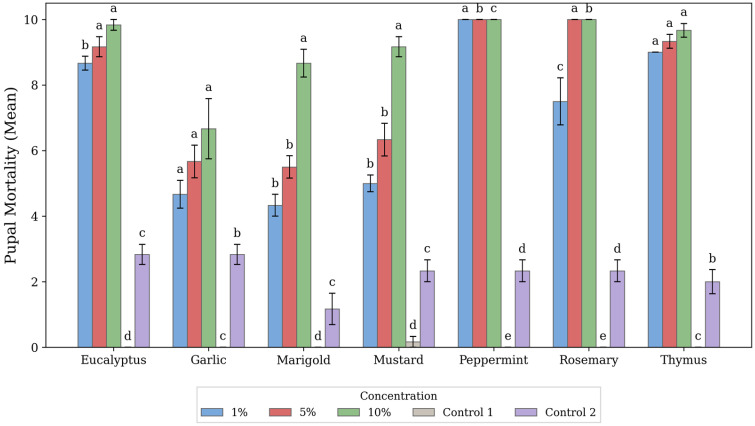
Mortality of *Cylas formicarius* pupae exposed to seven essential oils (eucalyptus, garlic, marigold, mustard, peppermint, rosemary, and thyme) at three concentrations (1%, 5%, and 10%). Control 1 = water; Control 2 = acetone. Bars represent mean pupal mortality ± standard error (SE) (n = 6). Different letters above the bars indicate significant differences among treatments, as determined by logistic regression followed by estimated marginal mean comparisons at *p* < 0.05.

The pupal mortality varied significantly among EO treatments and concentrations (Figure 5). Logistic regression analysis revealed significant effects of oil type (LR χ^2^ = 176.39; df = 6; *p* < 0.0001), concentration (LR χ^2^ = 1354.18; df = 4; *p* < 0.0001), and the interaction between oil type and concentration (LR χ^2^ = 117.37; df = 24; *p* < 0.0001). Pupal mortality increased with increasing EO concentration, with the 10% concentration consistently producing the highest mortality values across all evaluated oils. Peppermint and rosemary oils exhibited the strongest insecticidal effects, causing complete mortality at the 5% and 10% concentrations (10.0 ± 0.0). Thymus and eucalyptus oils also showed high pupal mortality at 10%, with means of 9.7 ± 0.2 and 9.8 ± 0.1, respectively.

Garlic and marigold oils demonstrated intermediate pupal mortality responses, increasing from 4.7 ± 0.4 and 4.3 ± 0.3 at 1% to 6.7 ± 0.9 and 8.7 ± 0.5 at 10%, respectively. Mustard oil also caused substantial pupal mortality at 10% (9.2 ± 0.3), although lower mortality levels were observed at lower concentrations. In contrast, both control treatments presented low mortality throughout the experiment. Control 1 showed values close to zero, whereas Control 2 ranged from 1.2 ± 0.4 to 2.8 ± 0.3. Overall, these results showed that increasing EO concentration significantly increased pupal mortality of *Cylas formicarius*, with peppermint, rosemary, thymus, and eucalyptus oils presenting the highest pupicidal activity.

### 3.5. Oviposition of Cylas formicarius

**Figure 6:**
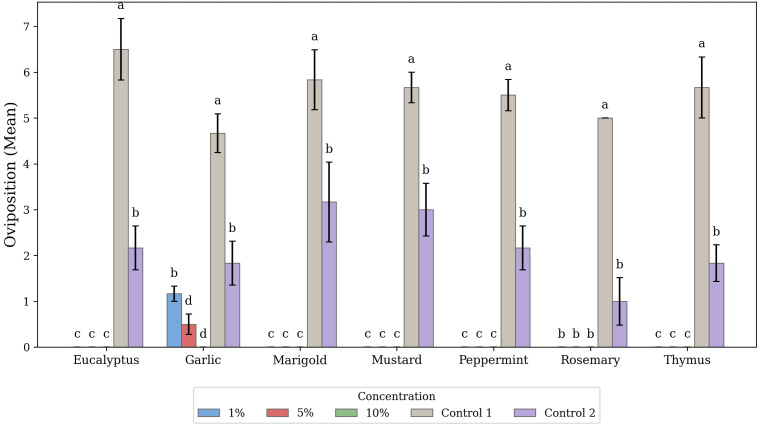
Oviposition of Cylas formicarius on sweet potato disks treated with seven essential oils (eucalyptus, garlic, marigold, mustard, peppermint, rosemary, and thyme) at three concentrations (1%, 5%, and 10%). Control 1 = water; Control 2 = acetone. Bars represent mean oviposition ± standard error (SE) (n = 6). Different letters above the bars indicate significant differences among treatments, as determined by logistic regression followed by estimated marginal mean comparisons at *p* < 0.05.

Oviposition was found to be significantly different among EO treatments and the evaluated concentrations (Figure 6). Logistic regression analysis revealed significant effects of oil type (LR χ^2^ = 37.98; df = 6; *p* < 0.0001) and concentration (LR χ^2^ = 804.38; df = 4; *p* < 0.0001) on oviposition response, whereas the interaction between oil type and concentration was not significant (LR χ^2^ = 19.33; df = 24; *p* = 0.7343). Overall, oviposition was reduced in all EO treatments compared with the controls. The control treatments presented the highest oviposition means among all evaluated oils. Control 1 exhibited the highest oviposition values, ranging from 4.7 ± 0.4 to 6.5 ± 0.7 eggs, whereas Control 2 ranged from 1.0 ± 0.5 to 3.2 ± 0.9 eggs.

The EO treatments, particularly at the 1, 5, and 10% concentrations, almost completely inhibited oviposition. Eucalyptus, marigold, mustard, peppermint, rosemary, and thymus oils resulted in complete or near-complete suppression of oviposition at all tested concentrations, with means close to zero. Garlic oil was the only treatment that showed a small amount of oviposition at the lower concentrations, with means of 1.2 ± 0.2 and 0.5 ± 0.3 eggs at 1% and 5%, respectively, whereas oviposition was absent at 10%.

### 3.6. Adult Mortality of Cylas formicarius

**Figure 7:**
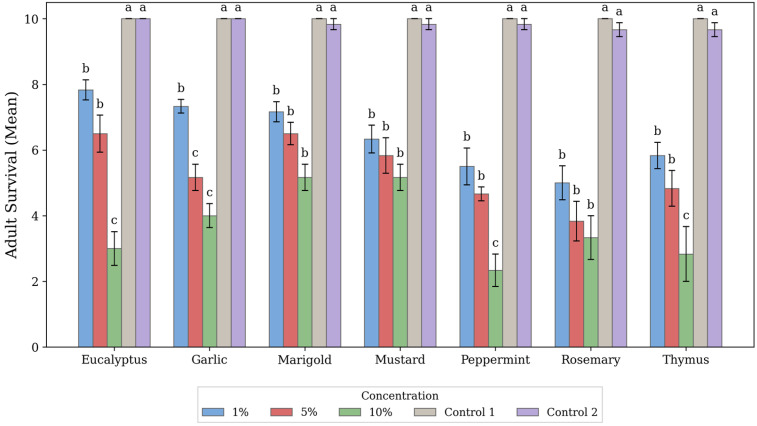
Adult survival of *Cylas formicarius* exposed to seven essential oils (eucalyptus, garlic, marigold, mustard, peppermint, rosemary, and thyme) at three concentrations (1%, 5%, and 10%). Control 1 = water; Control 2 = acetone. Bars represent mean adult survival ± standard error (SE) (n = 6). Different letters above the bars indicate significant differences among treatments, as determined by logistic regression followed by estimated marginal mean comparisons at *p* < 0.05.

Adult survival was found to be significantly different among EO treatments and concentrations (Figure 7). Logistic regression analysis revealed significant effects of oil type (LR χ^2^ = 37.98; df = 6; *p* < 0.0001) and concentration (LR χ^2^ = 804.38; df = 4; *p* < 0.0001) on adult survival, whereas the interaction between oil type and concentration was not significant (LR χ^2^ = 19.33; df = 24; *p* = 0.7343). Adult survival decreased as EO concentration increased, with the 10% concentration generally resulting in the lowest survival means across all evaluated oils. In contrast, both control treatments maintained the highest adult survival values, remaining close to complete survival throughout the experiment, with means near 10 insects per replicate.

Eucalyptus oil reduced adult survival from 7.8 ± 0.4 at 1% to 3.0 ± 0.5 at 10%, while garlic oil decreased survival from 7.3 ± 0.2 to 4.0 ± 0.4 across the same concentrations. Peppermint and rosemary oils produced some of the lowest adult survival means at 10%, with values of 2.4 ± 0.5 and 3.4 ± 0.7, respectively. Similarly, thymus oil reduced survival from 5.8 ± 0.5 at 1% to 2.9 ± 0.8 at 10%. Marigold and mustard oils exhibited intermediate effects on adult survival, although survival still decreased with increasing concentration. Overall, the results showed that increasing EO concentration significantly reduced adult survival of *C. formicarius*, with peppermint, rosemary, eucalyptus, and thymus oils showing the strongest effects at higher concentrations.

## 4. Discussion

This study determined that the evaluated EOs significantly affected feeding activity, larval and pupal mortality, adult survival, and oviposition of *C. formicarius*, with the intensity of the effects varying according to the type of oil and its concentration. In general, insecticidal activity increased with concentration, and the 10% concentration consistently produced the most pronounced biological responses across most evaluated parameters. Logistic regression analyses confirmed significant effects of EO type and concentration on larval mortality, pupal mortality, adult survival, and oviposition (*p* < 0.0001 in most analyses), indicating a clear concentration-dependent response. Increase in mortality (larvae, pupae, and adults) levels of the sweet potato weevil with higher concentrations suggests a concentration-dependent toxic effect of the tested essential oils on *Cylas formicarius*, likely resulting from greater exposure to bioactive compounds and enhanced physiological disruption at higher concentrations.

Volatile compounds in EOs exhibit direct toxicity, reducing pest activity [23,24,25]. The observed reductions in feeding indicate bioactive properties, such as feeding deterrence, mediated primarily by volatile compounds that disrupt olfactory function and, in some cases, cause direct toxicity [26,27,28]. Menthol and menthone in peppermint, and thymol and carvacrol in thyme, play important roles in behavioral modification and feeding reduction, while secondary constituents such as *p-cymene* and *γ-terpinene* may potentiate these effects [29]. The observed insecticidal effects are likely associated with the complex mixture of bioactive constituents present in the essential oils, as reported in the literature, while acknowledging that the specific compounds responsible for the activity were not verified in this study. The toxicity of EOs is often concentration-dependent, with higher concentrations increasing larval mortality, as observed in treatments with eucalyptus, peppermint, rosemary, and thyme. These oils affect larval physiological processes, influencing the nervous system, growth, and reproduction. Among the principal bioactive compounds, 1,8-cineole and α-pinene stand out in eucalyptus; menthol and menthone in peppermint; and 1,8-cineole, α-pinene, and camphor in rosemary, in addition to thymol and carvacrol in thyme [30,31]. Synergistic effects involving secondary constituents may further enhance deterrence and toxicity [32].

Similar patterns of stage-dependent susceptibility have previously been reported in other coleopteran insects exposed to botanical insecticides [33]. Similar effects have also been reported for botanical compounds against the sweet potato weevil, where strong repellent activity was observed for various EOs within the Lamiaceae family against *C. formicarius*, with repellency increasing with concentration [15]. Similarly, another study demonstrated that nanoemulsions based on plant extracts significantly reduced oviposition and increased adult mortality of *C. formicarius* under laboratory conditions [34]. Previous studies also reinforce the insecticidal potential of plant-derived compounds against *C. formicarius*; the insecticidal activity of *C. serrata* EO against adult sweet potato weevil was reported [14], while the biological activity of *Cleome viscosa* extracts against the same pest was observed under laboratory conditions [35]. Compared to previous studies, the results of the present work showed particularly high activity of peppermint oil, especially against the larval and pupal stages, reinforcing the potential of certain EOs as botanical insecticides.

EOs also interfere with pupal development, as demonstrated by oregano oil, which inhibited the metamorphosis of *Diaphania hyalinata* (Linnaeus) (Lepidoptera: Crambidae) [36]; furthermore, they inhibit oviposition through repellent properties, hormonal disruption, and the masking of host chemical signals [37,38]. Examples include lemon EO, which reduced oviposition by *Tetranychus urticae* (Koch) (Trombidiformes: Tetranychidae) by 99.15%, and basil oil, which exhibited 99% contact toxicity and deterrence against *Callosobruchus maculatus* (Fabricius) and *Callosobruchus chinensis* (Linnaeus) (Coleoptera: Chrysomelidae) [11,23]. *Mentha* species demonstrated strong oviposition deterrence against *Caryedon serratus* (Olivier) (Coleoptera: Chrysomelidae), with *Mentha spicata* completely inhibiting egg-laying at a concentration of 2% [39].

Despite the promising results observed, certain limitations of the present study must be acknowledged. This study was conducted under laboratory conditions using controlled-exposure bioassays; therefore, environmental factors such as temperature fluctuations, ultraviolet radiation, rainfall, volatilization, and interactions with plant surfaces were not evaluated and could influence the efficacy of the EOs under field conditions. Furthermore, the range of concentrations evaluated was relatively limited and did not allow for the establishment of complete dose–response curves or LC_50_ estimates for all developmental stages and biological parameters. Another limitation is that the persistence and residual activity of the EOs over time were not evaluated. Therefore, additional studies under semi-field and field conditions are necessary to validate the efficacy, stability, persistence, and practical applicability of these EOs in commercial sweet potato production systems. Nanoemulsified systems, in particular, may represent promising alternatives for enhancing the stability, bioavailability, and persistence of EO compounds under the agricultural conditions, as previously demonstrated [34]. Additional research, including semi-field and field evaluations, assessment of persistence under environmental conditions, non-target effects, compatibility with other management tactics, and economic feasibility, is needed before practical adoption for these EOs can be recommended.

## 5. Conclusions

This study demonstrated that the tested essential oil (EOs) treatments significantly affected adult survival, feeding activity, and oviposition of *Cylas formicarius* under laboratory conditions. Among the formulations evaluated, the 10% concentration consistently provided the greatest efficacy, reducing adult survival, feeding damage, and egg production while increasing mortality compared with untreated controls. These findings highlight the potential of EO-based formulations as environmentally sustainable alternatives to conventional insecticides for sweet potato weevil management. However, additional research is needed to identify the active compounds responsible for efficacy, evaluate formulation stability and persistence, and optimize application methods under semi-field and field conditions. Future studies should also assess long-term effectiveness and integration of EO-based products to support sustainable sweet potato production.

## Figures and Tables

**Table 1:** Literature-based review table of the main active compounds reported for the evaluated essential oils and their associated biological activities according to previous studies.

Essential Oil	Specific Producers	Plant Species	Active Compounds	Relevant Biological Activity	References
Eucalyptus	Kukka	*Eucalyptus globulus*	1,8-*cineole* (eucalyptol), α-*pinene*, limonene	Repellent, neurotoxic, feeding deterrent	[16]
Garlic	Sheer Essence	*Allium sativum*	Allicin, diallyl disulfide, diallyl trisulfide	Fumigant, insecticidal, feeding deterrent	[17]
Marigold	Deve Herbes	*Calendula officinalis*	Flavonoids, triterpenoids, sesquiterpenes	Moderate deterrent effect, growth inhibitor	[18]
White mustard	Talya	*Sinapis alba*	Allyl isothiocyanate, glucosinolates	Insecticidal, feeding deterrent, fumigant	[19]
Peppermint	Gya Labs	*Mentha piperita*	Menthol, menthone, pulegone	Strong repellent, feeding deterrent, neurotoxic	[20]
Rosemary	Kukka	*Rosmarinus officinalis*	1,8-*cineole*, camphor, α-*pinene*, borneol	Repellent, growth inhibitor, insect toxicant	[21]
Thyme	Hiqili	*Thymus gobicus*	Thymol, carvacrol, p-cymene	Strong feeding deterrent, repellent, neurotoxic	[22]

Each EO was diluted in analytical-grade acetone (≥99.5% purity) at different concentrations: 1%, 5%, and 10%. The solution preparation was carried out in a controlled environment using calibrated micropipettes (±0.1 µL accuracy) to ensure measurement precision.

**Table 2:** Matrix of essential oil treatments, concentrations, and solvent preparation used in the bioassays against *C. formicarius*.

Pure (100%) Essential Oil (Scientific Name)	Concentrations Tested (%)	Volume of EO Used (µL)	Solvent	Final Volume (mL)	
Eucalyptus (*Eucalyptus globulus*)	1, 5, 10	250, 1250, 2500	Acetone	25	
Garlic (*Allium sativum*)	1, 5, 10	250, 1250, 2500	Acetone	25	
Marigold (*Calendula officinalis*)	1, 5, 10	250, 1250, 2500	Acetone	25	
Mustard seed (*Sinapis alba*)	1, 5, 10	250, 1250, 2500	Acetone	25	
Peppermint (*Mentha piperita*)	1, 5, 10	250, 1250, 2500	Acetone	25	
Rosemary (*Rosmarinus officinalis*)	1, 5, 10	250, 1250, 2500	Acetone	25	
Thymus (*Thymus gobicus*)	1, 5, 10	250, 1250, 2500	Acetone	25	
Control 1		Distilled water	—	Water	—
Control 2	100% acetone	—		Acetone	—

## Data Availability

The original contributions presented in this study are included in the article. Further inquiries can be directed to the corresponding author.
